# IGF2BP3 as a Prognostic Biomarker in Well-Differentiated/Dedifferentiated Liposarcoma

**DOI:** 10.3390/cancers15184489

**Published:** 2023-09-09

**Authors:** Kyle D. Klingbeil, Jack Pengfei Tang, Danielle S. Graham, Serena Y. Lofftus, Amit Kumar Jaiswal, Tasha L. Lin, Chris Frias, Lucia Y. Chen, Manando Nakasaki, Sarah M. Dry, Joseph G. Crompton, Fritz C. Eilber, Dinesh S. Rao, Anusha Kalbasi, Brian E. Kadera

**Affiliations:** 1 Department of Surgery, Division of Surgical Oncology, University of California, Los Angeles David Geffen School of Medicine, Los Angeles, CA 90049, USAchrisfrias@mednet.ucla.edu (C.F.);; 2 Jonsson Comprehensive Cancer Center, University of California, Los Angeles, Los Angeles, CA 90095, USA; 3 Molecular, Cellular, and Integrative Physiology Interdepartmental PhD Program, University of California, Los Angeles, Los Angeles, CA 90095, USA; 4 University of California, Los Angeles David Geffen School of Medicine, Los Angeles, CA 90095, USA; 5 Department of Pathology & Laboratory Medicine, University of California, Los Angeles David Geffen School of Medicine, Los Angeles, CA 90095, USA; 6 Department of Medicine, Division of Hematology and Oncology, University of California, Los Angeles David Geffen School of Medicine, Los Angeles, CA 90095, USA; 7 Department of Medicine, Statistics Core, University of California, Los Angeles David Geffen School of Medicine, Los Angeles, CA 90095, USA; 8 Broad Stem Cell Research Center, University of California, Los Angeles, Los Angeles, CA 90095, USA; 9 Department of Radiation Oncology, Stanford Cancer Institute, Stanford School of Medicine, Stanford, CA 94305, USA

**Keywords:** soft-tissue sarcoma, IGF2BP3, IMP3, prognostic biomarker, liposarcoma, well-differentiated liposarcoma, dedifferentiated liposarcoma, tissue microarray, TCGA, gene microarray

## Abstract

**Simple Summary:**

Soft-tissue sarcoma (STS) is a rare cancer representing hundreds of unique subtypes. The prognosis of STS is heterogeneous, with few predictive biomarkers available beyond histologic subtype. IGF2BP3 is an RNA-binding protein that has recently been implicated in oncogenesis and tumor progression among various cancers. However, its association with STS has not been previously reported. In this study, we aimed to evaluate the expression and prognostic value of IGF2BP3 in STS. We found IGF2BP3 to be uniquely associated with poor survival among well-differentiated/dedifferentiated liposarcoma, a common subtype of STS, suggesting its role as a novel prognostic biomarker in this disease.

**Abstract:**

Background: Although IGF2BP3 has been implicated in tumorigenesis and poor outcomes in multiple cancers, its role in soft-tissue sarcoma (STS) remains unknown. Preliminary data have suggested an association with IGF2BP3 expression among patients with well-differentiated/dedifferentiated liposarcoma (WD/DD LPS), a disease where molecular risk stratification is lacking. Methods: We examined the survival associations of IGF2BP3 via univariate and multivariate Cox regression in three unique datasets: (1) the Cancer Genome Atlas (TCGA), (2) an in-house gene microarray, and (3) an in-house tissue microarray (TMA). A fourth dataset, representing an independent in-house TMA, was used for validation. Results: Within the TCGA dataset, IGF2BP3 expression was a poor prognostic factor uniquely in DD LPS (OS 1.6 vs. 5.0 years, *p* = 0.009). Within the microarray dataset, IGF2BP3 expression in WD/DD LPS was associated with worse survival (OS 7.7 vs. 21.5 years, *p* = 0.02). IGF2BP3 protein expression also portended worse survival in WD/DD LPS (OS 3.7 vs. 13.8 years, *p* < 0.001), which was confirmed in our validation cohort (OS 2.7 vs. 14.9 years, *p* < 0.001). In the multivariate model, IGF2BP3 was an independent risk factor for OS, (HR 2.55, *p* = 0.034). Conclusion: IGF2BP3 is highly expressed in a subset of WD/DD LPS. Across independent datasets, IGF2BP3 is also a biomarker of disease progression and worse survival.

## 1. Introduction

Recent work has implicated post-transcriptional gene regulation mediated by various factors, including RNA binding proteins, in cancer causation and maintenance. The RNA-binding protein, insulin-like growth factor 2 mRNA binding protein-3 (IGF2BP3), is overexpressed in a wide range of human cancers [1,2,3]. This includes a number of malignancies derived from all three primary germ layers, including epithelial malignancies and subtypes of hematolymphoid cancer [4,5,6,7,8,9,10].

Located on chromosome 7p15.3, *IGF2BP3* encodes an oncofetal protein expressed during embryogenesis, virtually absent in normal adult tissues, and strongly re-expressed in cancer cells [11]. IGF2BP3 belongs to the family of IGF2 mRNA-binding proteins, including the paralogs IGF2BP1 [12] and IGF2BP2 [13], and functions as a post-transcriptional regulator of gene expression. IGF2BPs bind to their target RNAs by recognizing specific RNA motifs in order to stabilize and enhance its translation, leading to the upregulation of oncogenic pathways [14,15,16]. Knockdown of IGF2BP3 inhibits cancer cell growth, motility, and the features of aggressive cancer in a variety of cancer subtypes [9,11,17,18]. Recent work has also demonstrated a functional role in vivo, and the critical role of the RNA-binding function of IGF2BP3 in oncogenesis [16,19].

As a prognostic biomarker, IGF2BP3 expression has been associated with disease progression and decreased survival in a growing list of cancer types, including non-small cell lung cancer [20], colorectal cancer [21], pancreatic ductal adenocarcinoma (PDAC) [22], clear cell renal cell carcinoma (RCC) [23,24], bladder carcinoma [25], breast cancer [26], and leukemia [16], among others [10,27,28,29,30]. These findings suggest a broad and pervasive role for this protein in cancer causation, but a role in soft-tissue sarcoma (STS) has not been extensively described to date.

STS represents a heterogeneous group of mesenchymal malignancies with poor outcomes. They comprise approximately 1% of adult and 15% of pediatric malignancies in the USA, with an annual incidence of 13,000 [31]. The heterogeneity of STS is vast, with over 100 described histologic subtypes that often include overlapping clinical and histopathological characteristics [32]. While an accurate diagnosis does guide tailored therapy, STS often recurs and mortality remains unacceptably high. Moreover, prognostic biomarkers are lacking.

Given the paucity of knowledge regarding the molecular landscape of STS and the need for improved prognostication, this study evaluates the expression and prognostic value of IGF2BP3 in a large cohort of STS subtypes across three unique patient datasets. We hypothesize that a subset of STS highly express IGF2BP3, and that IGF2BP3 overexpression predicts worse outcomes, including both overall (OS) and disease-free survival (DFS).

## 2. Methods

### 2.1. cBioportal Query and Cell Pathway Analysis

The cBio Cancer Genomics Portal (cBioportal) repository is an open-access platform that contains multi-omic data sets derived from more than 5000 patient tumor samples, and twenty research studies (http://cbioportal.org, accessed on 1 May 2022 [33,34]. We utilized the 2017 dataset from the Comprehensive and Integrated Genomic Characterization of Adult Soft Tissue Sarcomas that includes 206 samples of soft-tissue sarcoma, 50 of which represent dedifferentiated liposarcoma (DD LPS) [35]. Patient data including case ID, cancer type, detailed cancer type, mRNA expression z-scores relative to all samples (RNA Seq V2 RSEM), time of follow-up, and survival were exported for analysis. Samples with z-scores ≥ 1.0 were designated “IGF2BP3^+^”, and those < 1.0 designated “IGF2BP3^-^”. Genes positively co-expressed with IGF2BP3 with q values < 0.01 were inputted into the Metascape tool (www.metascape.org, accessed on 24 March 2023) to characterize the cell pathways upregulated in IGF2BP3^+^ tumors [36]. Additional plots automated by cBioportal were exported for inclusion in the current study.

### 2.2. Gene Expression Microarray Analysis

A previously generated in-house gene expression microarray was retooled for the purposes of the current study [37]. Expression values for individual genes were provided as log_10_ intensity ratios relative to a reference pool of labeled lipomatous tumors. An encrypted, clinically annotated patient database was used to match gene expression values to survival outcomes and tumor subtype. Well-differentiated (WD) and DD LPS samples with intensity ratios ≥ median value were designated “IGF2BP3^+^”, and those < median value were designated “IGF2BP3”.

### 2.3. Immunohistochemistry Staining and Quantification

Two in-house tissue microarrays of WD/DD LPS were sectioned and stained using Leica Bond RX under standard immunohistochemistry (IHC) protocols within UCLA’s Translational Pathology and Core Laboratory (TPCL). Briefly, automated detection was performed based on Protocol F using the Bond Polymer Refine Detection kit (Leica Biosystems, Wetzlar, Germany; Cat# DS9800). Heat-induced antigen retrieval was performed using the BOND Epitope Retrieval Solution 2, (Leica Biosystems, Cat#: AR9640) buffer for 20 min. Primary antibody was incubated for 60 min. Sections were incubated with DAKO EnVision+-HRP secondary antibody (Agilent Technologies, Santa Clara, CA, USA; Cat# K4003) for 10 min, followed by BOND Polymer Refine Detection DAB chromogen (Leica, Cat# DS9800). Tissue slides were probed with the following antibodies: IGF2BP3 [38] (Cell Marque, Rocklin, CA, USA; Cat# 433R, EP286, 1:25) and PDL1 (Abcam, Cambridge, United Kingdom; Cat# ab228462, 1:100). Tissue cores were annotated via pathological identification (path ID) using tissue maps, and manually verified by an expert pathology review (authors SD and MN). Tissue cores were classified as WD or DD based on histology of the tumor sample [39,40]. Missing or folded tissue cores were excluded. Quantification was obtained in a blinded manner using the HALO^®^ image analysis platform (Indica Labs, Albuquerque, NM, USA). Path IDs were then matched to an encrypted, clinically annotated database including median disease-free survival (mDFS) and overall survival (mOS) metrics. Replicate cores from the same path ID were averaged. Only primary tumor samples from each patient were used within subsequent survival analyses. ‘IGF2BP3^+^’ was designated for cores with ≥25% positivity, whereas ‘IGF2BP3^-^’ represented <25%, based on previously described protocols [41]. For samples with both transcriptional and translational IGF2BP3 expression data, co-expression was assessed using the Pearson correlation coefficient.

### 2.4. Cell Line Knockdown of IGF2BP3 using CRISPR/Cas9 Technology

LPS2 is a human liposarcoma cell line derived from DD LPS patient-derived xenografts at our institution [42]. IGF2BP3 was knocked down using CRISPR/Cas9 technology using a two-vector lentiviral system as previously described [19,43]. Briefly, LPS2 cells were stably transduced with a Cas9-P2A-EGFP transgene and sorted by flow cytometry to isolate GFP+ population. Next, lentiviruses prepared from a pLKO5.sgRNA.EFS.tRFP vector expressing non-targeting (NT) control sgRNA and Cr2 sgRNAs targeting IGF2BP3 were used to transduce Cas9-expressing cells. After 48 h, the transduced cells were sorted to isolate the +GFP/+RFP population. Sorted cells were expanded.

### 2.5. Western Blotting to Confirm IGF2BP3 Antibody Specifity

LPS2 cells were lysed with SDS buffer, and protein lysates were quantified using a BCA Protein Assay Kit (ThermoFisher, West Hills, CA, USA; Cat#23225) following the manufacturer’s instructions. Sample analysis was completed using the spectrophotometer settings at 562 nm on a Cytation 5 Imaging Reader (Agilent Technologies, Santa Clara, CA, USA). Protein lysates were electrophoresed using 4–12% Bis-Tris gels. Resolved proteins were transferred to a PVDF membrane. The following primary antibodies were used: IGF2BP3 (Cell Marque, Cat# 433R, EP286, 1:1000), IGF2BP3 (MBL International Corp, Woburn, MA, USA; Cat# N009P, 1:1000) and Beta-actin (Cell Signaling Technology, Danvers, MA, USA; Cat# 3700S, 1:2000), which served as a loading control. Bound antibodies were visualized using ImobilonTM Western (Millipore Corporation, Billerica, MA, USA).

### 2.6. Statistical Analysis

Statistical analyses were performed using R Version 4.3.1 and GraphPad Prism 9.5.1 (GraphPad Software, Boston, MA, USA). Data presented as mean ± SEM for continuous numerical data, unless otherwise noted. A one-way analysis of variance (ANOVA) test was used to assess the difference between the means of >2 groups, followed by pair-wise comparisons using the Bonferroni test. A two-tailed Student’s *t* test was used for comparisons of two groups, unless otherwise specified. The Fisher Exact test was used to compare categorical variables. Pearson’s correlation estimate was applied to determine associations between variables of interest. A Wilcoxon signed rank test was used to compare IGF2BP3 positivity between matched pairs of initial and recurrent tumor samples. Survival associations were assessed using univariate and multivariate Cox proportional hazard regression models, displayed as hazard ratios (HR). The Kaplan–Meier estimate was used to determine median survival. Goodness-of-fit for the regression models was assessed using the concordance (C) statistic and the Akaike Information Criterion (AIC), where ΔAIC < 2 shows no difference between models; 2 ≤ ΔAIC < 7 is some evidence that the model with a lower AIC is better; and ΔAIC ≥ 7 is strong evidence that the model with a lower AIC is better [44]. Confidence intervals (CI) of 95% were used, and a *p* value < 0.05 was considered statistically significant.

## 3. Results

**Figure 1:**
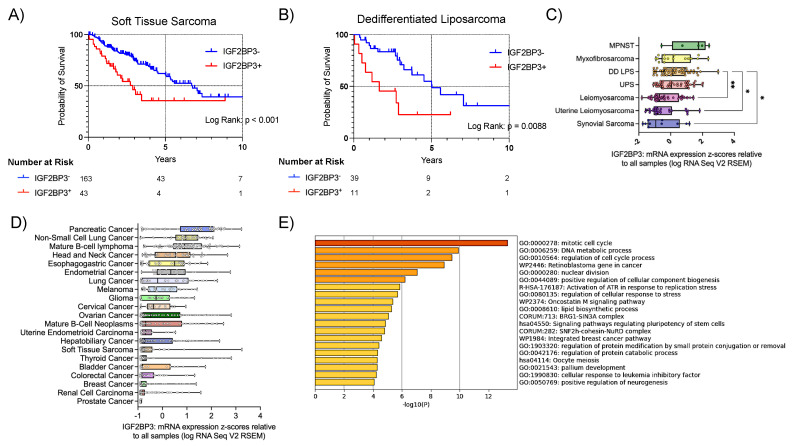
Worse survival uniquely associated with *IGF2BP3* expression in dedifferentiated liposarcoma among various subtypes of sarcoma. Figure 1 Legend: (**A**) Kaplan-Meier curve comparing overall survival for all soft-tissue sarcoma samples (*n* = 206) included in the Cell 2017 TCGA database, stratified by IGF2BP3 mRNA expression. mOS: 1.6 vs. 5.0 years. Log-Rank *p* < 0.001. (**B**) Kaplan–Meier curve comparing overall survival for all DD LPS samples (*n* = 50) included in the Cell 2017 TCGA database, stratified by IGF2BP3 mRNA expression. mOS: 1.6 vs. 5.0 years. Log-Rank *p* = 0.0088. (**C**) Comparison of *IGF2BP3* expression among various subtypes of soft-tissue sarcoma included within the Cell 2017 TCGA database, *n* = 206; data displayed as box and whisker plot, with min, mean, max and all points shown. One-way ANOVA, *p* < 0.0001. Significant comparisons by Bonferroni’s test are shown, (* *p* < 0.05, ** *p* < 0.01). (**D**) IGF2BP3 RNA expression levels for cancers included in the pan-cancer analysis of whole genomes (TCGA, Nature 2020), ordered by median expression. Data are displayed as box and whisker plot with min, max, and all points shown. (**E**) Metascape pathway enrichment analysis displaying the top 20 cell pathways upregulated in IGF2BP3^+^ tumors, ordered by −log_10_ (*p* value). DD LPS, dedifferentiated liposarcoma. mOS, median overall survival. MPNST, malignant peripheral nerve sheath tumor. UPS, undifferentiated pleomorphic sarcoma.

IGF2BP3 has garnered significant attention due to its frequency of overexpression in many cancer types, lack of expression in normal adult tissues, and the association of expression levels with poor prognosis [45]. To evaluate IGF2BP3′s role in STS, we began by comparing IGF2BP3 overexpression with overall survival (OS) using the TCGA dataset of STS [35]. IGF2BP3^+^ tumors portended worse survival compared to IGF2BP3^−^ (mOS 6.76 years vs. 2.88 years, *p* < 0.001) (Figure 1A). This cohort was then stratified by STS subtype to explore whether prognostication of *IGF2BP3* expression would be maintained. We found DD LPS uniquely portended poor survival for IGF2BP3^+^ tumors (mOS 5.00 vs. 1.6 years, *p =* 0.0088) (Figure 1B), whereas the remaining tumor subtypes showed no difference in survival (Figure S1A–C).

We then compared *IGF2BP3* expression levels among STS subtypes, and identified DD LPS among the highest expressing subtypes of STS (Figure 1C). Comparing STS to a TCGA cohort of pan-cancer [46], unsurprisingly, we found a range of IGF2BP3 expression across different cancer types (Figure 1D). Next, we evaluated the gene expression of known targets of IGF2BP3 among IGF2BP3^+^ tumors to explore their potential role in STS. *HMGA2* was identified as the highest differentially expressed gene, following *IGF2BP3*, in IGF2BP3^+^ tumors (Figure S2A). This finding is important, as *HMGA2* has previously been implicated as a target of IGF2BP3 [11,30]. Interestingly, *HMGA2* is commonly amplified in WD/DD LPS [47]. Using a similar approach for IGF2BP3^+^ tumors in only DD LPS samples, expression of *HMGA2* and *CDK6*, both of which have been implicated as targets of IGF2BP3 [19,30], was positively correlated with *IGF2BP3* expression (Figure S2B–D). We then utilized a Metascape pathway enrichment analysis and found *IGF2BP3* expression was associated with multiple pathways involved in cell proliferation and replication (Figure 1E). Together, these data suggest a prognostic association with *IGF2BP3* expression in STS, specifically implicating its role in oncogenesis and tumor progression for DD LPS.

**Figure 2:**
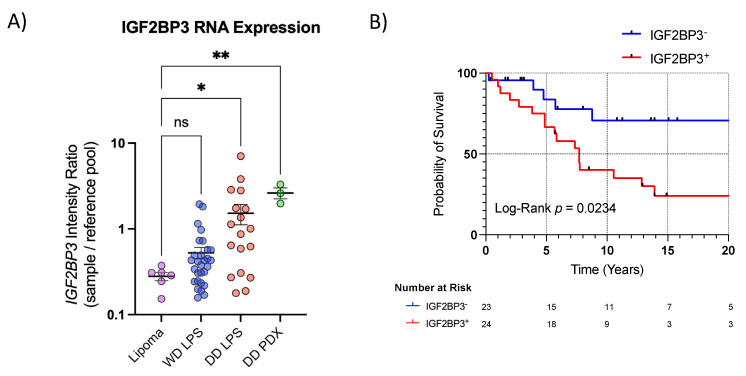
*IGF2BP3* transcriptional expression highest in DD LPS and associated with worse survival in WD/DD LPS. Figure 2 Legend: (**A**) *IGF2BP3* transcriptional expression viewed as scatter plot. Lipoma (*n* = 6), WD LPS (*n* = 29), DD LPS (*n* = 18), DD LPS patient-derived xenograft (PDX) (*n* = 3). ANOVA *p* < 0.001, with multiple comparisons by Bonferroni test displayed, (* *p* < 0.05, ** *p* < 0.01). (**B**) *IGF2BP3* transcriptional expression was associated with worse overall survival in WD/DD LPS, HR 2.47 (1.1,5.3) (mOS 7.7 vs. 21.5 years). Log-Rank *p* = 0.0234. DD PDX, dedifferentiated liposarcoma patient-derived xenografts. NS, not significant. WD LPS, well-differentiated liposarcoma.

To confirm these initial findings, we retooled an in-house gene expression microarray of 47 WD/DD LPS patient samples (Table S1) [37]. DD LPS displayed higher expression compared to the benign lipoma control, *p* = 0.0078, whereas WD LPS *IGF2BP3* expression was not significant, *p* = 0.999 (Figure 2A). After stratifying WD/DD LPS patients by *IGF2BP3* expression, IGF2BP3^+^ was associated with worse survival in WD/DD LPS, mOS 7.7 vs. 21.5 years, *p* = 0.0234 (Figure 2B).

**Figure 3:**
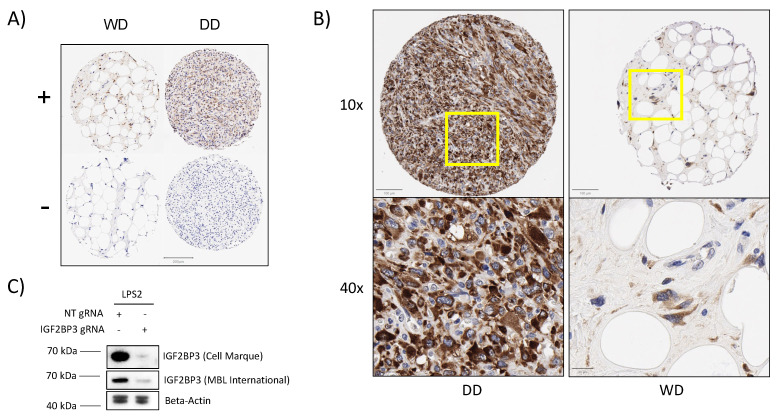
IGF2BP3 translational expression validated as a prognostic biomarker using an independent cohort of WD/DD LPS. (**A**) Representative IGF2BP3^+^ and IGF2BP3^−^ core samples of WD and DD histologic subtypes following IGF2BP3 immunohistochemistry staining. (**B**) Representative IGF2BP3+ cores from WD and DD samples demonstrating strong homogenous cytoplasmic expression. “10×” and “40×” signify microscopic magnification. The yellow square indicates the location of 40x inset. (**C**) Western blot of human liposarcoma cell line, LPS2, using a previously validated IGF2BP3 antibody by MBL International, and an IGF2BP3 antibody by Cell Marque. The original images of the Western blots are shown in Figure S4.

Next, we assessed whether IGF2BP3 protein expression predicted survival in WD/DD LPS. To accomplish this, we used an in-house tissue microarray (TMA) containing 97 cores of WD LPS (*n* = 32) and DD LPS (*n* = 65) (Table 1). Patients most commonly presented in the fifth decade of life, tumor location was mostly retroperitoneal, and median follow-up was 6.82 years, (range 0–24.8). IHC confirmed IGF2BP3 expression in both subtypes of LPS (Figure 3A). DD LPS demonstrated higher cell positivity, *p* = 0.0054 (Figure S3A) and three DD LPS PDX models previously developed at our institution included in the tissue microarray maintained high IGF2BP3 positivity (Figure S3C). IGF2BP3 staining was homogenous and localized to the cytoplasm in positive samples, similar to previously validated cancers (Figure 3B) [22,38].

To confirm the binding specificity of the IGF2BP3 antibody, and validate the prognostic value of IGF2BP3 in future studies, we performed Western blotting of a human liposarcoma cell line, LPS2, following CRISPR-cas9 mediated IGF2BP3 knockdown [19,43]. LPS2 demonstrated high protein expression of IGF2BP3 that was lost when IGF2BP3 gRNA was expressed, confirming the binding specificity of the antibody (Figure 3C). This specificity was similar to that of a previously validated antibody for IGF2BP3 [19].

**Figure 4:**
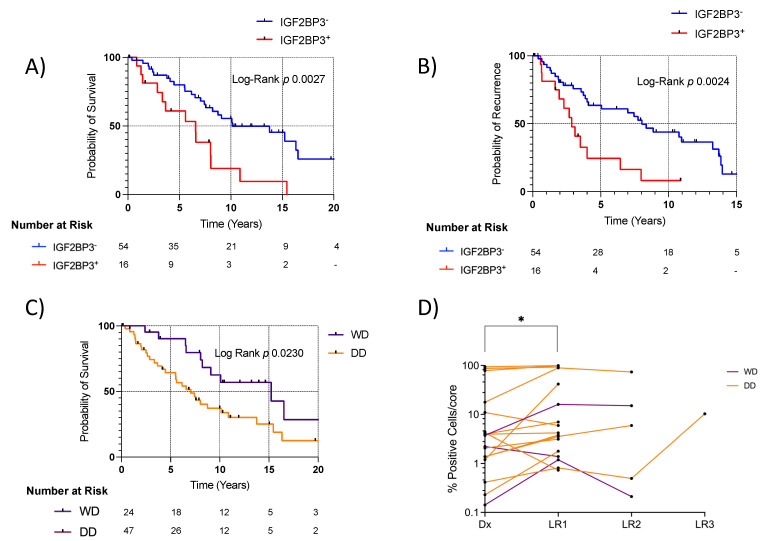
Stratification by IGF2BP3 protein expression is associated with survival in WD/DD LPS. Figure 3, Legend: (**A**) IGF2BP3^+^ tumors were associated with worse survival in WD/DD LPS, mOS: 6.6 vs. 10.1 years. HR 2.6 (CI 1.1, 6.2). Log-rank *p* = 0.0027. C-statistic 0.59, AIC = 256.5. (**B**) IGF2BP3^+^ tumors also associated with recurrence in WD/DD LPS, mDFS: 2.9 vs. 8.0 years. HR 2.6 (CI 1.1, 6.0). Log-rank *p* = 0.0024. (**C**) Overall survival by LPS histological subtype at initial presentation, mOS: 7.0 (DD) vs. 15.2 years (WD). HR 2.2 (CI 1.2, 4.2). Log-rank *p* = 0.023. C-statistic 0.60, AIC = 259.2. (**D**) For patients with both primary and recurrent tumor cores, IGF2BP3 positivity increased after first LR, using a Wilcoxon signed rank test, *p* = 0.03. (* *p* < 0.05) Histologic subtype did not change between initial presentation and recurrence in the included patient samples. LR, local recurrence. HR, hazard ratio. mDFS, median disease-free survival.

When stratifying WD/DD LPS samples by IGF2BP3 protein expression, IGF2BP3^+^ was again associated with worse survival (mOS: 3.7 vs. 13.8 years, *p* < 0.0001) (Figure 4A). IGF2BP3^+^ was also associated with recurrence (mDFS: 3.7 vs. 13.8 years, *p* = 0.005) (Figure 4B). Comparing these findings to the LPS histological subtype, we found a mOS of 7.0 (DD) vs. 15.2 years (WD), *p* = 0.023 (Figure 4C). There was some evidence that stratification by IGF2BP3 expression was more strongly associated with worse survival than stratification by histologic differentiation status, at ΔAIC = 2.7. For patients with both primary and recurrent tumor samples included in the TMA, IGF2BP3 positivity appeared to increase after recurrence (*p* = 0.03), with a clear positive trend in subsequent recurrences (Figure 4D).

**Figure 5:**
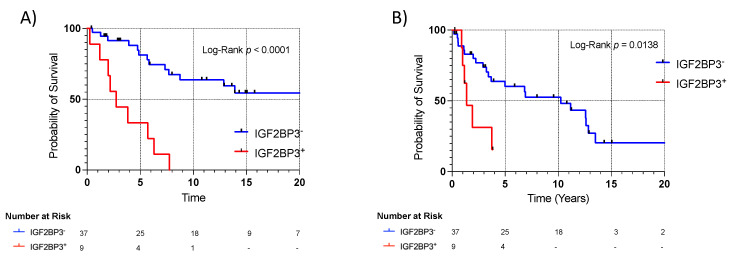
IGF2BP3 translational expression validated as prognostic biomarker using an independent cohort of WD/DD LPS. Figure 4 Legend: (**A**) IGF2BP3 expression displayed overall survival in WD/DD LPS, mOS: 2.74 vs. 21.2 years. HR 4.89 (CI 1.3, 18.0), log-rank *p* < 0.0001. (**B**) IGF2BP3 expression associated with recurrence in WD/DD LPS, mDFS: 1.4 vs. 10.3 years. HR 2.8 (CI 0.77, 10.5), log-rank *p* = 0.0138.

To validate IGF2BP3 protein expression as a prognostic biomarker in WD/DD LPS, we analyzed a second, independent TMA of WD/DD LPS. IGF2BP3^+^ displayed worse survival (mOS: 2.7 vs. 14.9 years, *p* < 0.0001) (Figure 5A). IGF2BP3^+^ was also associated with recurrence (mDFS: 2.7 vs. 8.0 years, *p* = 0.005) (Figure 5B). For WD/DD LPS samples included in both the gene expression microarray and TMA (*n* = 43), IGF2BP3 protein and mRNA expression was highly correlated r^2^ = 0.69, (Pearson correlation coefficient, *p* < 0.001). After adjusting for clinically relevant variables, the multivariate Cox model demonstrated that IGF2BP3^+^ remained an independent risk factor for OS, (HR 2.55, *p* = 0.034) (Table 2).

In addition to modulating tumor-intrinsic factors, IGF2BP3 has been implicated in the immunomodulation of the tumor microenvironment, from the upregulation of immune checkpoint inhibitors to the polarization of tumor-associated macrophages (TAMs) towards an immunosuppressive phenotype [25,48,49]. In STS, there is significant heterogeneity in the number and populations of immune cell infiltrates, even among tumors of the same histologic subtype [50]. So, as a final exploratory analysis, we investigated the co-expression of IGF2BP3 and PDL1 by IHC. PDL1 positivity was not significantly different between histologic subtypes (*p* = 0.696), or when comparing primary vs. recurrent samples (*p* = 0.400), (Figure S5A,B). No association between IGF2BP3 and PDL1 expression was found (*p* = 0.781), (Figure S5C).

## 4. Discussion

In the present study, IGF2BP3 overexpression predicted worse survival in STS, consistent with its known association with poor prognosis in a growing list of cancer types [10,16,20,21,22,23,24,25,27,28,30]. Additionally, prognostication by IGF2BP3 overexpression appeared to be specific to DD LPS, which was studied across multiple patient platforms and validated by an additional unique dataset. IGF2BP3^+^ was also shown to be an independent risk factor for OS in the multivariate model.

LPS is a malignant tumor of adipocyte lineage, and is among the more common STS subtypes, accounting for 15–20% of cases [51]. Classically, there are five histologic subtypes that vary in their molecular landscape, clinical behavior, and treatment sensitivity: well-differentiated liposarcoma (WD LPS), dedifferentiated liposarcoma (DD LPS), myxoid liposarcoma, pleomorphic liposarcoma, and myxoid pleomorphic liposarcoma [52]. Molecularly, WD LPS and DD LPS share amplified segments of chromosome region 12q13-15, which contains a number of cancer-related genes implicated in tumorigenesis, including *MDM2* and *CDK4* [53,54]. Surgical resection with negative margins remains the mainstay treatment. Radiation therapy is typically considered for high-grade STS, such as DD LPS [55,56,57], whereas its role in WD LPS is more limited [58]. Systemic chemotherapy is doxorubicin with or without ifosfamide in the first-line setting, and reserved for unresectable or metastatic disease [59]. Recurrence of both WD and DD LPS remains unacceptably high, especially if located in the retroperitoneum [60]. Conventional histologic stratification alone, however, fails to fully capture disease heterogeneity. To improve prognostication, several groups have proposed nomograms using a combination of clinical and histologic data in order to guide prognostication and treatment [61,62,63]. Inclusion of novel prognostic biomarkers, such as IGF2BP3, may therefore further personalize patient care to optimize treatment selection.

The mechanistic role of IGF2BP3 in WD/DD LPS remains poorly understood. Previous work characterizing the role of IGF2BP3 in other cancers may offer insight into a shared pathway. For instance, in melanoma, IGF2BP3 promotes migration and invasion through direct regulation of HMGA2 transcripts [30]. HMGA2 is an oncofetal protein involved in cell proliferation, neoplastic transformation, and tumor invasion [64]. We found *HMGA2* expression to be highly associated with IGF2BP3 in both STS and WD/DD LPS. This may represent one mechanism by which IGF2BP3 promotes cancer progression; however, we acknowledge the role of IGF2BP3 is likely multifaceted.

As an RNA binding protein, the function of IGF2BP3 is intimately linked to the cancer cells’ unique transcriptional program. IGF2BP3 target genes and the mechanisms by which IGF2BP3 contributes to oncogenesis can vary widely by cell type, from regulation of cell cycle-related genes in B-ALL and gliomas to migration and invasion genes in PDAC [19,65,66]. In colorectal carcinoma, IGF2BP3 has been shown to activate the MEK1/ERK signaling pathway and promote anti-apoptotic pathways through the stabilization of Bcl-2 and Bcl-xL transcripts [67,68]. In clear-cell RCC, IGF2BP3 contributes to cancer progression and metastasis through activation of the NfKB pathway [69]. In each instance, IGF2BP3 may act to amplify a particular oncogenic program specific to the cancer type. This is akin to its role in mixed-lineage leukemia, wherein IGF2BP3 expression, induced by the MLL-AF4 leukemogenic pathway, positively regulates MLL-AF4 transcriptional targets, creating a feedforward process that drives cancer progression [16].

IGF2BP3 has previously been associated with genes involved in M-phase and cell cycle regulation in pilocytic/pilomyxoid astrocytomas. Curiously, in these tumors, unlike other markers of cell cycling, only IGF2BP3 was found to correlate with disease progression [29]. This highlights a possible role for IGF2BP3 in mitotic and cell cycle regulation, and suggests that IGF2BP3 may be a more sensitive marker for dividing cells, capable of detecting smaller ranges of differences in cycling frequencies. In this study, we showed IGF2BP3 expression was associated with M-phase and cell cycle pathways.

We also noted in our enrichment analysis that IGF2BP3 expression associates with signaling pathways regulating pluripotency of stem cells. Prior studies in triple-negative breast cancer have implicated IGF2BP3 in the genesis and function of cancer stem cells, through direct regulation of SLUG transcripts, which in-turn regulates Sox2 [26]. Thus, IGF2BP3 may have an important role within cancer stem cells that warrants further study.

As immunotherapy for STS continues to be investigated, attention to novel biomarkers that predict response is warranted. Here, we explored canonical PDL1 expression across WD/DD LPS samples and coupled its expression with IGF2BP3. While PDL1 expression was overall low, and no association with IGF2BP3 was present, our findings provide the groundwork for further exploration into other immune cell populations within the tumor microenvironment. In kidney cancer, NK cell-mediated immunity is thought to be the most important mechanism through which cancer cells evade the immune system [9]. Additionally, efforts in STS have demonstrated a multifaceted role of B lymphocytes in the tumor microenvironment [70]. Future exploration of IGF2BP3 in this context is therefore warranted.

Further questions regarding the role of IGF2BP3 in LPS remain. The most common subtypes of LPS, WD/DD, demonstrated a survival difference with IGF2BP3 overexpression; however, other genetically diverse subtypes of LPS were not evaluated. Future studies may consider a more expansive focus of these remaining subtypes, and also further exploration of additional STS subtypes to answer whether IGF2BP3 has utility as a prognostic biomarker beyond WD/DD LPS. The limitations of the current study include its retrospective approach, exploratory nature, and selection bias, as all patients with transcriptional and/or translational data underwent surgical resection. Tissue biopsy may also be considered in future prospective studies to assess the role of IGF2BP3 in predicting treatment response, and to better capture all patients suffering from this rare disease process.

Despite the current progress in understanding the role of IGF2BP3 as a prognostic biomarker in STS, further research to explore the molecular mechanisms underlying this observation is warranted. Efforts to define the IGF2BP3-regulated transcriptome in STS may reveal oncogenic transcripts that could serve as therapeutic targets. Comparing transcripts in STS with previously defined malignancies will be critical to furthering our understanding of the conserved post-transcriptional mechanisms that regulate oncogenic proliferation and differentiation to ultimately guide the next generation of therapy.

## 5. Conclusions

IGF2BP3 expression may offer improved stratification for patients diagnosed with WD/DD LPS beyond histologic classification, thereby personalizing care to more accurately inform clinical decision-making. The clinical utility of IGF2BP3 as a prognostic biomarker warrants further investigation in larger, prospective study cohorts.

## Figures and Tables

**Table 1:** Patient characteristics within the tissue microarray cohorts. Table 1, Legend: Median follow-up (range) for all patients included in the initial TMA: 6.82 years (0–24.8), and validation TMA: 6.19 (0.25–56.4). Categorical variables are described as *n* (frequency) for each group. A two-sided Fisher’s exact test was used to compare statistical significance. Continuous variables are described as average (standard deviation). A two-sided unpaired Student’s *T* test was used to compare statistical significance. ^~^ Continuous variable; * *p* values < 0.05. RP, retroperitoneum.

	Initial TMA			Validation TMA		
Covariate	IGF2BP3^+^ (*n* = 16)	IGF2BP3^−^ (*n* = 54)	*p* value	IGF2BP3^+^ (*n* = 9)	IGF2BP3^−^ (*n* = 37)	*p* value
Age (years) ^~^	57.6 (13.2)	60.9 (13.5)	0.23	64.9 (14)	61.6 (14)	0.53
Gender—Male	8 (50)	28 (51.9)	0.99	4 (44.4)	16 (43.2)	0.99
Histology DD WD	10 (62.5) 6 (37.5)	35 (64.8) 19 (35.2)	0.99	9 (100) 0 (0)	12 (32.4) 25 (67.6)	0.086
Tumor Location RP Extremity	15 (93.8) 1 (6.2)	54 (100) -	0.23	1 (11.1) 8 (88.9)	25 (67.6) 12 (32.4)	0.006 *
Tumor Size (cm) ^~^	27.3 (9.9)	26.9 (11.2)	0.48	22.7 (10.0)	18.9 (9.4)	0.28
Neoadjuvant Chemotherapy	1 (6.3)	6 (11.1)	0.99	1 (11.1)	3 (8.1)	0.99
Adjuvant Chemotherapy	2 (12.5)	7 (13.0)	0.99	5 (55.6)	7 (18.9)	0.039 *
Radiation Therapy	5 (31.3)	14 (25.9)	0.75	7 (77.8)	21 (56.8)	0.45
Tumor Recurrence	11 (68.8)	31 (57.4)	0.56	6 (66.7)	22 (59.5)	0.99
Death	13 (81.3)	25 (46.3)	0.021 *	9 (100)	18 (48.6)	0.0062 *

**Table 2:** Multivariate Cox regression model for overall survival. Table 2, Legend: Multivariate Cox Regression Model using clinicopathologic data from the initial TMA cohort. Candidate covariates were chosen based on clinical relevancy. Data are presented as a hazard ratio and 95% confidence interval. C-statistic = 0.70, AIC = 255.3. * *p* values < 0.05.

Covariate	Reference	HR	(95% CI)	*p* Value
Age	-	1.05	1.02, 1.08	0.002 *
Gender—Female	Male	0.86	0.4, 1.86	0.709
Histology—DD	WD	1.34	0.58, 3.11	0.495
Tumor Size	-	0.98	0.95, 1.02	0.365
Neoadjuvant Chemotherapy—No	Yes	2.08	0.46, 9.28	0.339
Adjuvant Chemotherapy—No	Yes	1.39	0.55, 3.52	0.489
Radiation Therapy—No	Yes	1.21	0.58, 2.53	0.609
Tumor Recurrence—Yes	No	1.02	0.46, 2.29	0.958
Expression—IGF2BP3^+^	IGF2BP3^−^	2.55	1.07, 6.04	0.034 *

## Data Availability

The following data were previously presented at the American Association for Cancer Research (AACR) Annual Meeting 2022 in New Orleans, Louisiana.

## Supplementary Materials

The following supporting information can be downloaded at: https://www.mdpi.com/article/10.3390/cancers15184489/s1, Figure S1: Survival associations of IGF2BP3 expression among various non-LPS subtypes of soft-tissue sarcoma included in the TCGA; Figure S2: Exploratory analysis of co-expression in IGF2BP3+ samples included in the Soft Tissue Sarcoma TCGA Database; Figure S3: IGF2BP3 protein expression maintained among established WD/DD LPS PDX-derived cell lines; Figure S4: Raw images of SEM and LPS2 Western blots; Figure S5: WD/DDLPS displays low PDL1 expression and does not correlate with IGF2BP3 expression; Table S1: Patient characteristics within the mRNA microarray cohort.
