# Supplementary figures and images for: IGF2BP3 as a Prognostic Biomarker in Well-Differentiated/Dedifferentiated Liposarcoma
Source: Cancers (Basel). 2023 Sep 9;15(18):4489. doi: 10.3390/cancers15184489 (PMC10526143; doi:10.3390/cancers15184489)

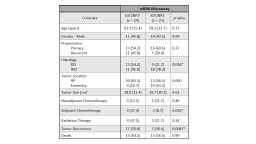

Supplement: Supplementary file 1 [file cancers-15-04489-s001.zip › cancers-2557382-supplementary/docProps/thumbnail.jpeg]

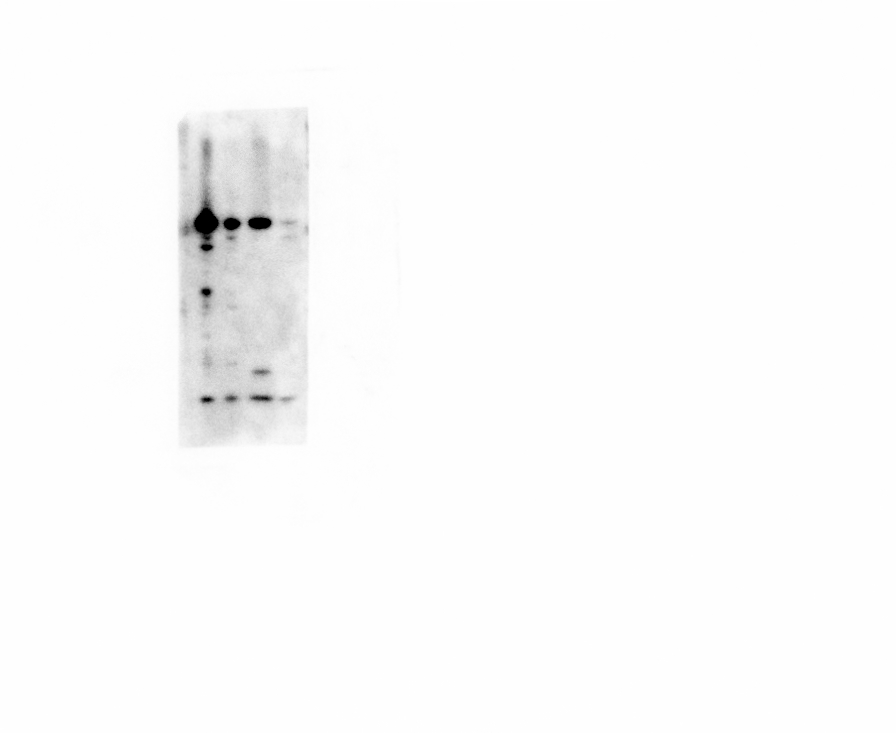

Supplement: Supplementary file 1 [file cancers-15-04489-s001.zip › cancers-2557382-supplementary/ppt/media/image13.png]

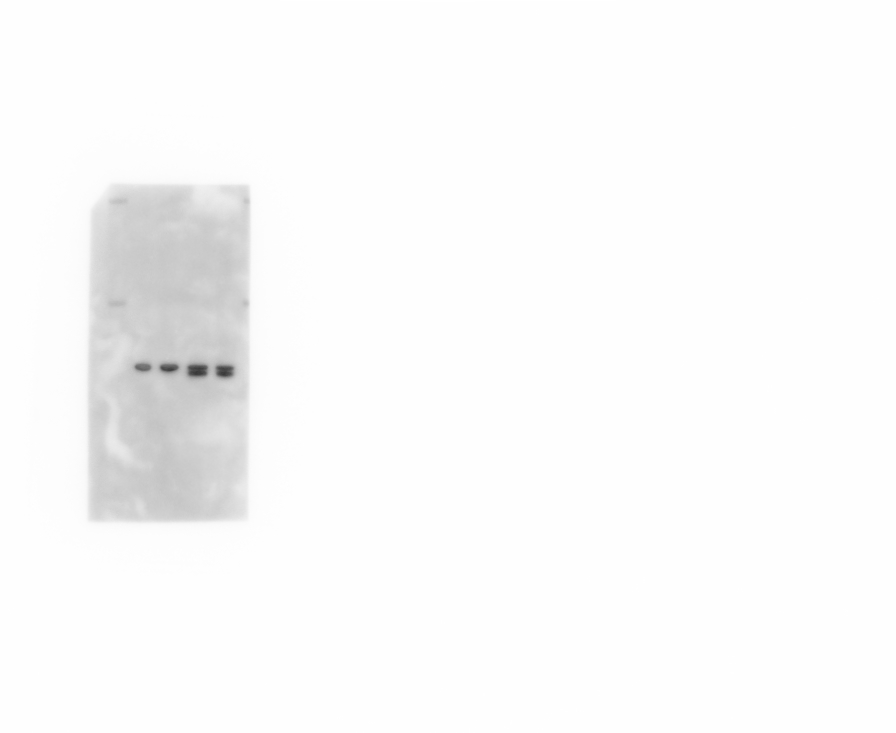

Supplement: Supplementary file 1 [file cancers-15-04489-s001.zip › cancers-2557382-supplementary/ppt/media/image14.png]

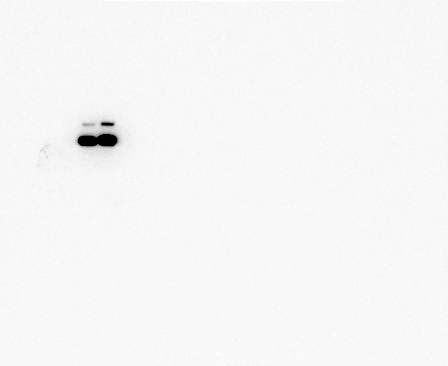

Supplement: Supplementary file 1 [file cancers-15-04489-s001.zip › cancers-2557382-supplementary/ppt/media/image15.png]

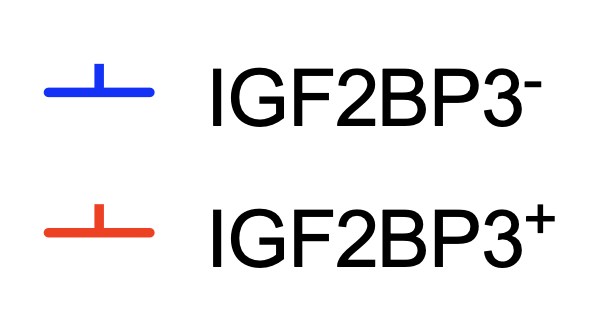

Supplement: Supplementary file 1 [file cancers-15-04489-s001.zip › cancers-2557382-supplementary/ppt/media/image4.png]

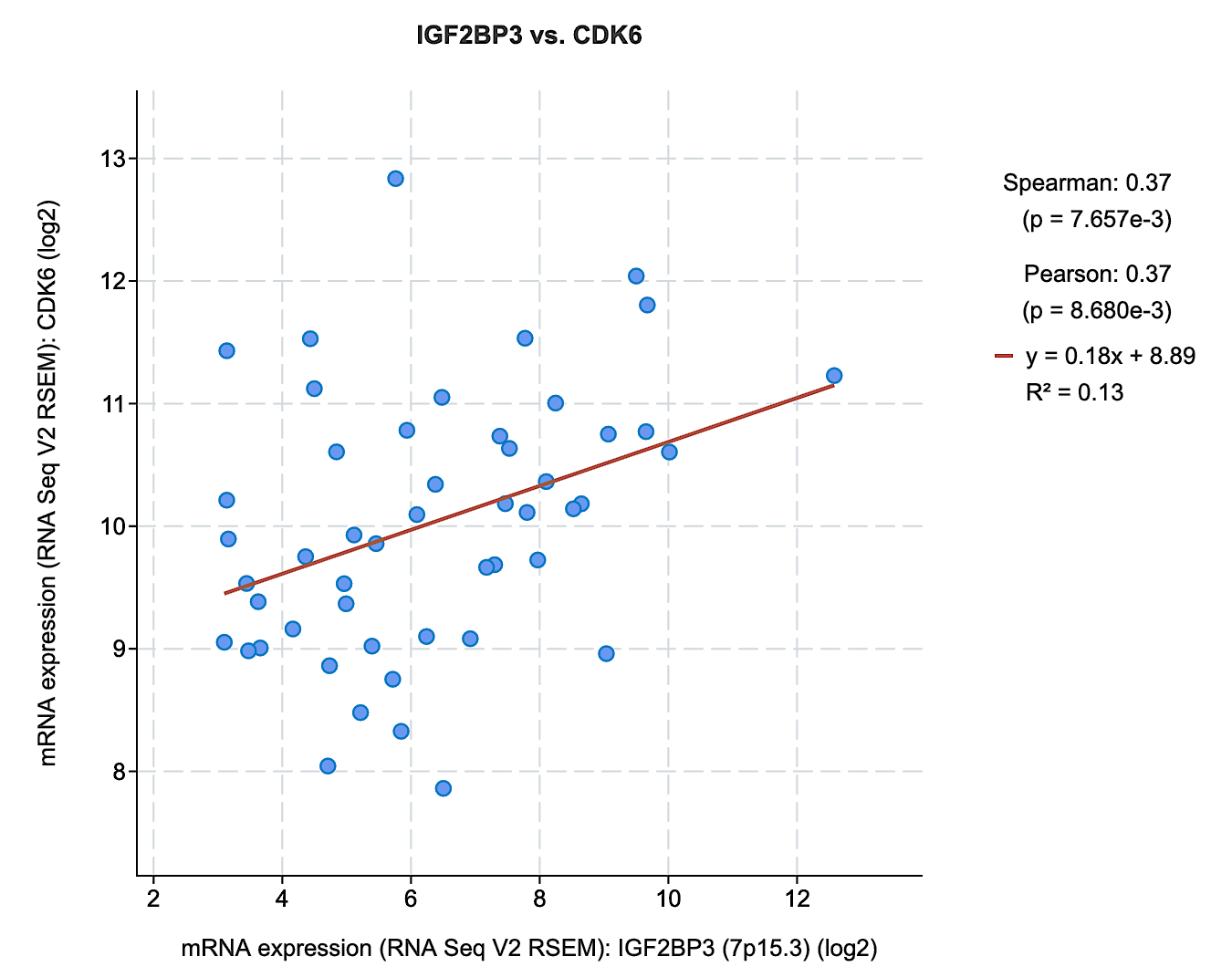

Supplement: Supplementary file 1 [file cancers-15-04489-s001.zip › cancers-2557382-supplementary/ppt/media/image5.png]

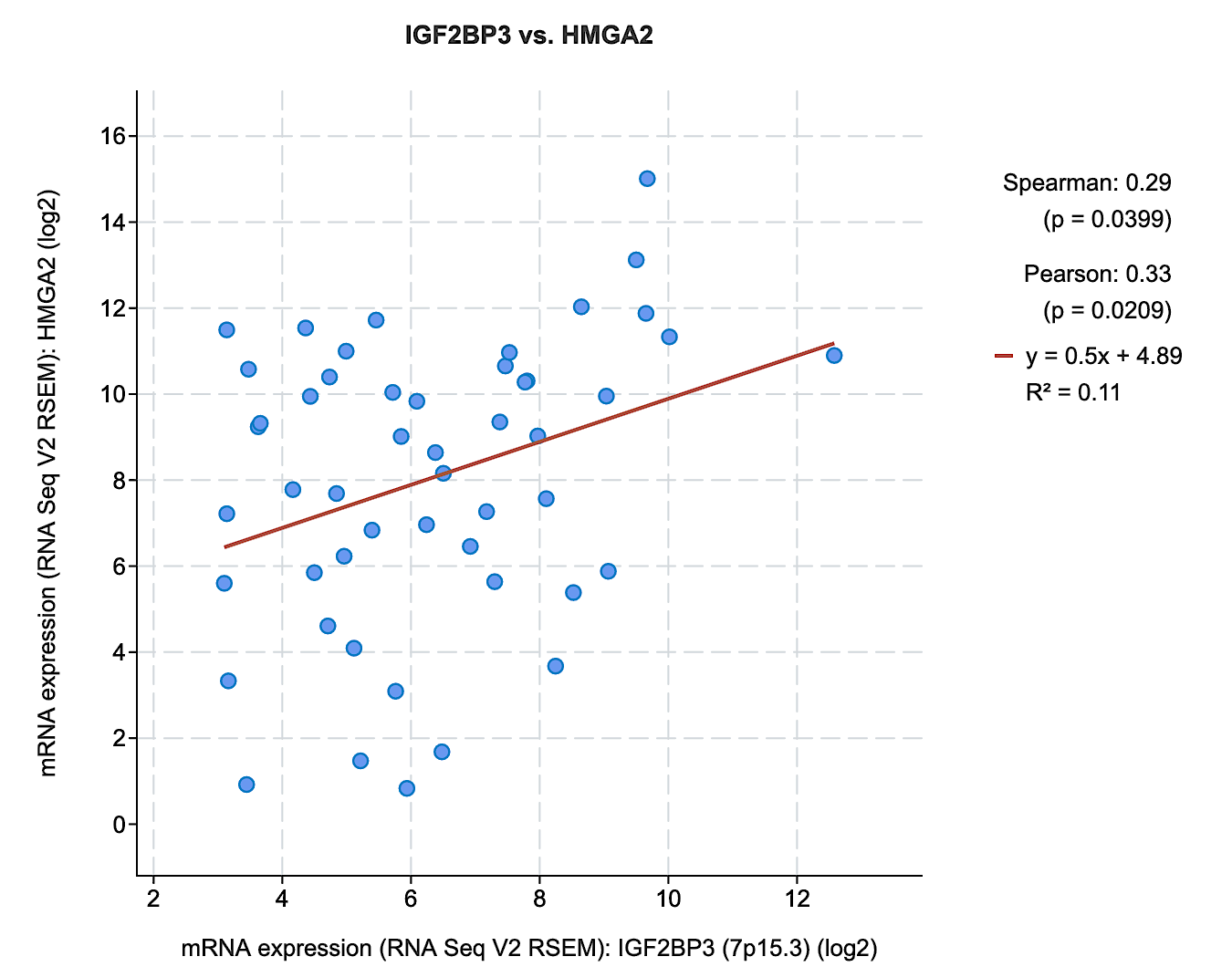

Supplement: Supplementary file 1 [file cancers-15-04489-s001.zip › cancers-2557382-supplementary/ppt/media/image6.png]

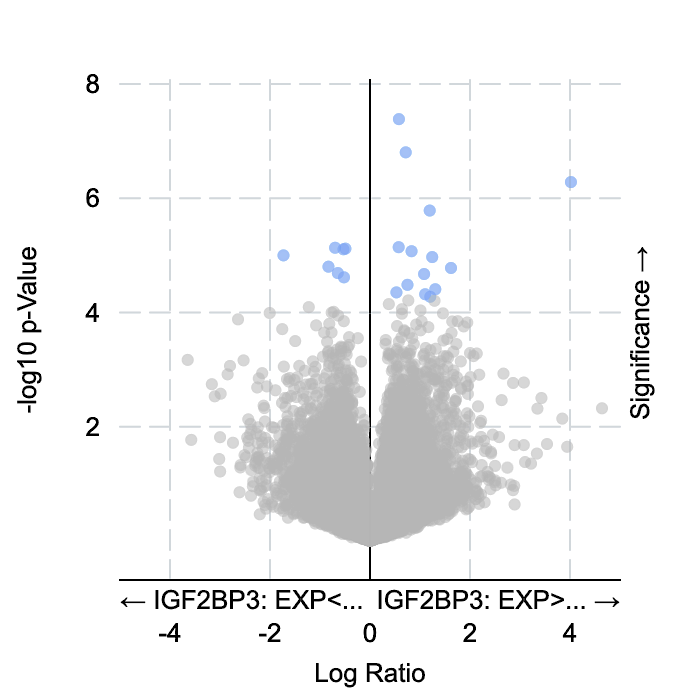

Supplement: Supplementary file 1 [file cancers-15-04489-s001.zip › cancers-2557382-supplementary/ppt/media/image7.png]

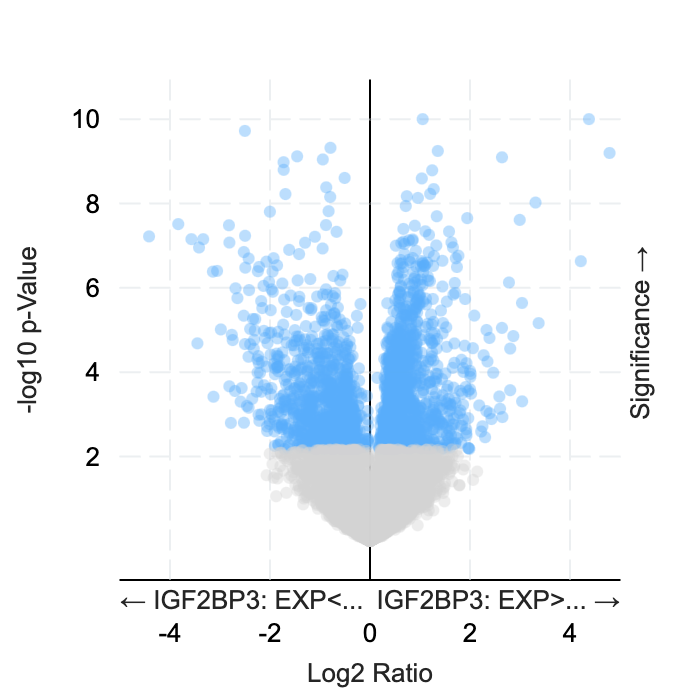

Supplement: Supplementary file 1 [file cancers-15-04489-s001.zip › cancers-2557382-supplementary/ppt/media/image8.png]

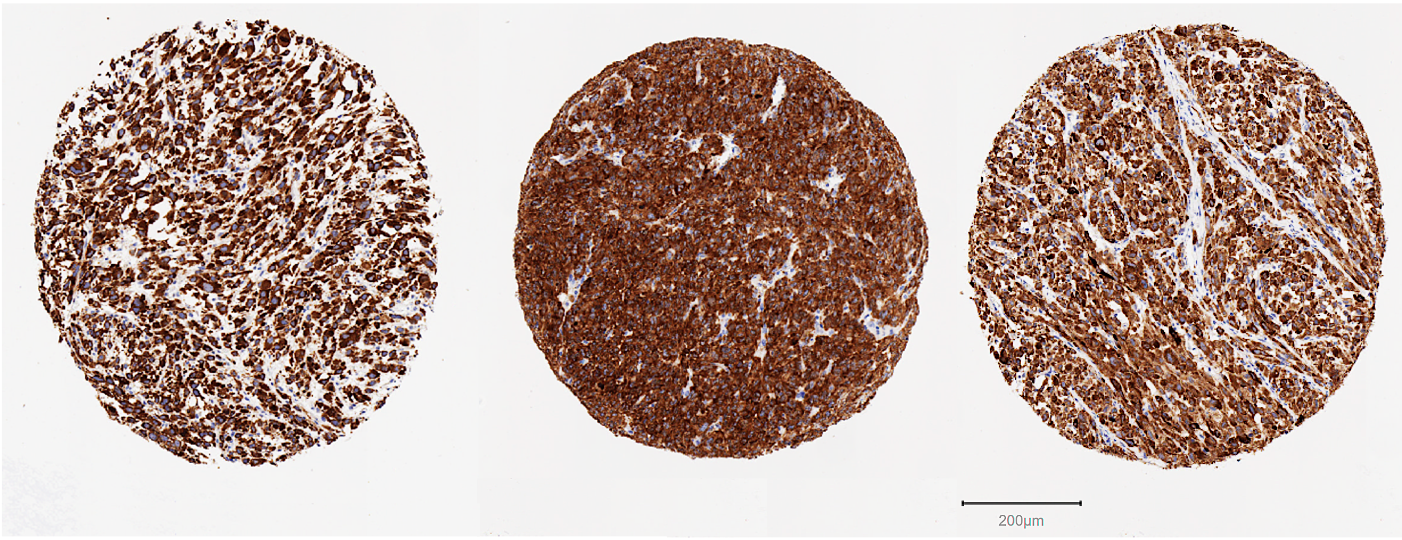

Supplement: Supplementary file 1 [file cancers-15-04489-s001.zip › cancers-2557382-supplementary/ppt/media/image9.png]
